# Pandan–vanilla rotation mitigates *Fusarium* wilt disease in vanilla: insights from rhizosphere microbial community shifts

**DOI:** 10.3389/fmicb.2025.1496701

**Published:** 2025-06-18

**Authors:** Shan Hong, Yizhang Xing, Jinming Yang, Qingyun Zhao, Fan Su, Huifa Zhuang, Hui Wang, Zhuangsheng Wu, Yisong Chen

**Affiliations:** ^1^Sanya Institute, Hainan Academy of Agricultural Sciences/Hainan Key Laboratory of Vegetable Biology, The Institute of Vegetables, Hainan Academy of Agricultural Sciences, Sanya, Hainan Province, China; ^2^State Key Laboratory of Plant Genomics, Institute of Genetics and Developmental Biology, Chinese Academy of Sciences, Beijing, China; ^3^Hainan Seed Industry Laboratory, Sanya, Hainan Province, China; ^4^National Nanfan Research Institute (Sanya), Chinese Academy of Agricultural Sciences, Sanya, Hainan Province, China; ^5^Spice and Beverage of Institute, Chinese Academy of Tropical Agricultural Sciences/Key Laboratory of Genetic Resources Utilization of Spice and Beverage Crops, Ministry of Agriculture and Rural Affairs/Hainan Provincial Key Laboratory of Genetic Improvement and Quality Regulation for Tropical Spice and Beverage Crops, Wanning, Hainan Province, China; ^6^The Sanya Institute of Nanjing Agricultural University, Nanjing Agricultural University, Sanya, Hainan Province, China

**Keywords:** crop rotation, vanilla, pandan, microbial community, rhizosphere microbiome

## Abstract

**Introduction:**

Vanilla monoculture often leads to *Fusarium* wilt disease, affecting the industry globally.

**Methods:**

Here, we evaluated the effects of vanilla–black pepper, –pandan, and –sweet rice tea rotations (i.e., growing vanilla in soil previously planted with these crops) on *Fusarium oxysporum* abundance and rhizosphere microbial communities using real-time quantitative PCR and high-throughput sequencing.

**Results:**

Pandan rotation, in particular, reduced disease incidence to 17% and decreased *F. oxysporum* copy numbers; sweet rice tea showed similar suppressive effects. Crop rotation significantly increased fungal diversity and richness. Different cropping systems, including fallow, monoculture and crop rotation, significantly influenced fungal and bacterial community development, with cropping system and rotated crops being the main drivers of rhizosphere community assembly. The black pepper and pandan rotations specifically enriched certain fungal OTUs, such as OTU1_*Thermomyces*, OTU37_*Arthrobotrys*, and OTU18_*Arthrobotrys*, which serve as biomarkers for the presence of *F. oxysporum*. After pandan rotation, microbial interactions within the rhizosphere intensified, with notable enrichment of core bacterial taxa, including OTU22_*Nitrosospira*, OTU56_*Lacibacterium*, and OTU178_*Actinospica*. Soil pH was identified as a significant factor influencing microbial community assembly. The fungal community structure, along with core OTU22_*Nitrosospira* and soil pH, was pivotal in curbing pathogen growth, explaining 25.19%, 8.61%, and 20.45% of the variance, respectively.

**Conclusion:**

This study revealed that incorporating pandan into crop rotation may effectively alleviate soil-borne diseases during vanilla production.

## Introduction

Vanilla (*Vanilla planifolia*), a member of the Orchidaceae family and the *Vanilla* genus, is an herbaceous perennial vine in tropical and subtropical regions (Minoo et al., 2008; Koyyappurath et al., 2016). It is highly acclaimed for its distinctive aroma and flavor. However, it is prone to *Fusarium* wilt disease caused by the soil-borne pathogen *Fusarium oxysporum* f. sp. *vanillae*, which results in severe replanting issues, reduced yield, and inferior quality (Pinaria et al., 2010; Mosquera-Espinosa et al., 2022). Our previous study elucidated how different durations of monoculture affect the soil microbial community structure and composition in vanilla cultivation systems. We discovered that long-term monoculture intensifies the incidence of *Fusarium* wilt disease (DI) in vanilla due to alterations in the soil microbial community, particularly due to decreases in beneficial microbes and the growth of *F. oxysporum* pathogens (Xiong et al., 2015).

Rhizosphere microbial communities are essential for enhancing the disease resistance and stress tolerance of crops (Trivedi et al., 2020). The diversity of these communities is intricately connected to the overall health of crops (Pedrinho et al., 2024). Research has shown that high diversity in functional soil microbes can improve crop resistance to diseases and stress tolerance by facilitating nutrient uptake and the synthesis of antimicrobial substances (Romero et al., 2023). Rhizosphere microbes, particularly fungi, are crucial for forming and maintaining soil structure, suppressing pathogens, and enhancing crop disease resistance (Yarzábal Rodríguez et al., 2024). Beneficial fungi compete with pathogens for nutrients and living space in the soil (Wang and Kuzyakov, 2024). By rapidly occupying specific ecological niches in the rhizosphere and forming stable microbial community structures, they limit the invasion and growth of pathogens (Li et al., 2022). Upon interacting with plant roots, some fungi can activate plant systemic resistance mechanisms (Rabari et al., 2023). Additionally, there is a complex synergistic relationship between fungi and bacteria in the inhibition of pathogens. Bacteria can promote the growth of fungi, while the metabolic products produced by fungi can enhance the antibacterial capabilities of bacteria (Napitupulu, 2025). Similar to those in the rhizosphere, mycosphere microbial communities are also effective in alleviating fungal pathogens of mushrooms (Yu et al., 2025), demonstrating the shared principles of microbiome biocontrol capabilities. Furthermore, agricultural management strategies such as crop rotation significantly influence the composition and functionality of soil microbial communities, thereby positively affecting crop health and productivity (Yang et al., 2020; Longepierre et al., 2021).

Crop rotation is an effective strategy for managing soil health through the modulation of the abundance and diversity of the soil microbial community, ensuring the health of both the soil and crops (Iheshiulo et al., 2023). This approach is environmentally friendly and economically viable for controlling soil-borne diseases (Zhou et al., 2023). We developed crop rotation systems, including black pepper (*Piper nigrum* L.) rotation with vanilla and coffee (*Coffea* L.) rotation with vanilla, and reported that black pepper–vanilla rotation significantly stimulated beneficial fungal genera such as *Trichoderma* and *Penicillium* (Xiong et al., 2016). However, as black pepper is a perennial crop, creating additional crop rotation systems is essential for addressing vanilla replanting problems and achieving high economic returns on limited arable land, which is crucial for the sustainable development of the vanilla industry (Yang et al., 2024).

Core rhizosphere microbes, as key indicator species, regulate microbial community structures through plant–microbe interactions or through interactions among microbes (Toju et al., 2018; Hong et al., 2023). Our preliminary research indicated that in long-term vanilla monoculture systems, changes in fungal communities are closely related to the suppression of *Fusarium* wilt disease, with the core disease-suppressing microbe *Mortierella* being predominant. It accounted for 37% of the total fungal sequences and may serve as an indicator of *Fusarium* wilt disease suppression in vanilla (Xiong et al., 2017). Moreover, soil physicochemical properties, particularly pH changes, significantly impact microbial community structure and core rhizosphere microbe abundance (Hong et al., 2020). Soil pH significantly regulates microbial community structure and pathogen suppression efficacy through both direct and indirect pathways. In terms of direct effects, pH modulates the catalytic activity and substrate-binding capacity of microbial extracellular enzymes (e.g., urease and phosphatase) and intracellular enzymes by altering their ionization states. Regarding indirect mechanisms, neutral to slightly alkaline environments (pH 6.5–7.5) enhance the quorum sensing efficiency of antagonistic microbial communities (e.g., *Pseudomonas* spp.), which restricts pathogens' iron acquisition or suppresses the expression of virulence genes by modulating the synthesis of siderophores and antibiotics. Concurrently, pH-driven shifts in nutrient availability further shape microbial competitive interactions. Hence, integrating soil physicochemical property analysis with microbial community analysis is essential for understanding how crop rotation effectively suppresses soil-borne *Fusarium* wilt disease in vanilla (Hermans et al., 2020; Philippot et al., 2024).

Previous research on the role of crop rotation in enhancing disease resistance in vanilla has been focused mainly on perennial crops, with an emphasis on disease suppression mechanisms mediated by soil fungal communities. However, there is a significant gap in the analysis of soil bacterial communities and in the comprehensive examination of the interplay between soil microbes and physicochemical properties. To fill this gap and meet evolving industrial needs, in this study, we identified two characteristic tropical spice crops—pandan (*Pandanus amaryllifolius*) and sweet rice tea (*Strobilanthes tonkinensis Lindau*)—for the first time for crop rotation with vanilla. Both are herbaceous plants with 6-month production cycles. They require minimal field management, thus keeping labor costs low, yet yield an output valued at $7,500 to $9,600 per hectare, making them popular among local farmers. Accordingly, soil that had been under rotation with pepper, pandan, and sweet rice tea for 1 year was collected and replanted with vanilla. Using qPCR combined with high-throughput sequencing, we focused on assessing the disease-suppressive effects of the rotations on vanilla and impact on the rhizosphere soil microbial community postrotation under greenhouse conditions. In this research, we aim to provide theoretical insights for developing effective crop rotation strategies to suppress soil-borne *Fusarium* wilt disease in vanilla cultivation.

## Materials and methods

### Experiment description

The pot experiment was conducted from May 2023 to April 2024 in the greenhouse of the Spice and Beverage Research Institute at the Chinese Academy of Tropical Agricultural Sciences. Greenhouse conditions were maintained at 22–25°C during the daytime, 20–25°C at nighttime, with 72% relative humidity and 75% light transmittance. The soil used in the pot experiments was naturally infested soil collected from a vanilla plantation at the Spice and Beverage Research Institute, located in Longxing Town, Wanning City, Hainan Province, China (110°19′E−110°22′E, 18°12′N−18°16′N). This site has been continuously cultivated with vanilla for over 10 years, with an annual *Fusarium* wilt disease incidence of 65%. The soil texture was classified as sandy loam, with the following physicochemical properties: pH 5.58, organic matter (OM) 25.82 g-kg^−1^, alkali-hydrolyzable nitrogen (AN) 100.32 mg-kg^−1^, available phosphorus (AP) 20.46 mg-kg^−1^, and available potassium (AK) 150.46 mg-kg^−1^.

Prior to the experiment, all soil was transported to the greenhouse and sieved through a 2-mm mesh. The soils were under rotation with black pepper, pandan, and sweet rice tea. After 1 year of crop rotation, the soils were collected and vanilla was planted. The soils cropped with black pepper, pandan, and sweet rice tea were designated as black pepper–vanilla rotation (H), pandan–vanilla rotation (B), and sweet rice tea–vanilla rotation (C), respectively. Soils with vanilla monoculture were labeled as X, while fallow soils served as controls (CK). Notably, this study specifically focused on evaluating the disease suppression capacity of vanilla replanted after crop rotation. Therefore, the post-rotation soils were excluded from subsequent analysis.

The vanilla variety tested was Mexican vanilla (*Vanilla planifolia Andrews*). Vanilla is propagated asexually, so we planted 60 cm long mature stems from high-quality parent plants. The experiment was based on a randomized block design. Each treatment was replicated three times to ensure the reliability of the results. Each replicate consisted of six pots, with three plants per pot. Each pot was filled with 15 kg of soil, and the pot dimensions were 32 cm × 25 cm, ensuring sufficient space between each plant and independence to avoid potential cross-contamination. Each pot received 90 g of organic fertilizer, which was carefully mixed with the soil using a small shovel to ensure even distribution. The organic fertilizer had a pH of 7.51 and contained 1.33% nitrogen (N), 2.54% P_2_O_5_, and 1.23% K_2_O. The chemical fertilizer application was based on traditional agricultural practices. Soil samples were collected from the rhizosphere compartment after 12 months of cultivation. Notably, this experiment was focused on evaluating the disease suppression effects of vanilla replanted after crop rotation. Therefore, the soils after crop rotation were not subjected to further research. The disease incidence (DI) of vanilla due to *Fusarium* wilt was statistically assessed on the basis of typical symptoms and was calculated as the percentage of affected plants out of the total surveyed (Xiong et al., 2016).

### Soil sampling and DNA extraction

Seven pots were randomly selected from each treatment, and the three vanilla plants in each pot were combined to form one sample. Each treatment resulted in seven composites soil samples. The vanilla plants were carefully removed from the soil, immediately preserved in an ice box at 4°C, and transported promptly to the laboratory. The soil closely associated with the root system was dislodged by gently shaking the plant roots, and this soil was designated rhizosphere soil (Chen et al., 2020). In total, 35 rhizosphere soil samples were obtained. Each sample was accurately weighed to 0.4 grams for DNA extraction using the PowerSoil DNA Isolation Kit from QIAGEN/MoBio (MoBio Laboratories, Carlsbad, CA, USA). The extraction was performed according to the manufacturer's protocol, ensuring meticulous and error-free execution of the procedure. The extracted DNA samples were subsequently stored at −70°C for future analysis.

### Soil physicochemical analysis

In accordance with our previous report (Hong et al., 2020), soil pH was measured in a soil–water suspension (1:2.5 w/v) via a glass electrode attached to a pH meter after shaking for 30 min. The organic matter (OM) content was determined using the potassium dichromate titration method, where potassium dichromate oxidizes organic carbon, and the organic matter content is calculated by titrating the dichromate remaining. Available nitrogen (AN) was measured using the alkaline diffusion method, where sodium hydroxide is used to treat the soil, and the resulting ammonia is absorbed by boric acid, followed by titration with standard acid. Available phosphorus (AP) was extracted with sodium bicarbonate and determined by the molybdenum blue method. Available potassium (AK) was extracted with ammonium acetate and measured using flame photometry.

### Quantification of *F. oxysporum*

The overall abundance of *F. oxysporum* in the rhizosphere soil of vanilla was ascertained via real-time quantitative PCR (qPCR), employing the pathogen-specific primer sets AFP308R (CGAATTAACGCGAGTCCCAAC) and ITS1F (CTTGGTCATTTAGAGGAAGTAA) as previously described (Xiong et al., 2017). A standard curve was constructed via a serial dilution method using a plasmid harboring the internal transcribed spacer (ITS) region of *F. oxysporum*. Quantitative PCR was performed on the Applied Biosystems QuantStudio 7 Flex, following a standardized protocol for template amplification. The PCR mixture had a final volume of 20 μl, containing 10 μl of SYBR^®^ Premix Ex Taq™ (2 × ), 0.4 μl of each primer (final concentration of 10 μM), 0.4 μl of ROX Reference Dye II (50 × ), 1 μl of DNA template (~20 ng/μl), and nuclease-free water to the full volume. The performance of PCR amplification was assessed by examining the dissociation curve and determining the amplification efficiency. Triplicate analyses were conducted for each sample, and the data were log-transformed to express the results as log-transformed copy numbers per gram of dry soil.

### Sequencing of amplicons

The V4 region of the 16S rRNA gene from soil bacteria was amplified using the primers 520F (AYTGGGYDTAAAGNG) and 802R (TACNVGGGTATCTAATCC; Caporaso et al., 2011). For soil fungi, the ITS1 region was amplified with the primers ITS5F (GGAAGTAAAAGTCGTAACAAGG) and ITS1R (GCTGCGTTCTTCATCGATGC; Schoch et al., 2012). Unique barcodes/linkers and adapters were incorporated during the amplification process to prepare samples for sequencing. The PCR mixture, totaling 25 μl, included 5 × reaction buffer (5 μl), 5 × GC buffer (5 μl), 10 μM primers (1 μl), template DNA (2 μl), 100 mM dNTPs (5 μl), and nuclease-free water to the full volume. The PCR amplification parameters consisted of initial denaturation at 98°C for 2 min, followed by 28 to 30 cycles of denaturation at 98°C for 15 s, annealing at 55°C for 30 s for bacteria and at 50°C for 30 s for fungi, with extension at 72°C for 30 s.

Following PCR amplification, the resulting products were purified using the QIAquick PCR Purification Kit (QIAGEN, Germany). The concentration of the purified DNA samples was measured using a Qubit^®^ 2.0 fluorometer (Invitrogen, USA). The sequencing libraries were prepared from equimolar pooled samples using the NEBNext^®^ Ultra™ DNA Library Prep Kit for Illumina (New England Biolabs, UK), after which they were read for sequencing. The quality of the constructed libraries was assessed and verified via an Agilent 2,100 Bioanalyzer (Agilent Technologies, USA) and a KAPA Library Quantification Kit (KAPA Biosystems, USA). After library construction, the library was loaded onto the flow cell of the Illumina MiSeq sequencer. The flow cell surface underwent bridge PCR amplification to form numerous DNA clusters. During sequencing, the sequencer sequentially added nucleotides labeled with fluorescent markers to the DNA chains. Each incorporated nucleotide emitted a specific fluorescent signal, and the DNA sequence was determined by detecting the order of these signals. The entire process from library construction to sequencing was executed and completed by Shanghai Personal Biotechnology Co., Ltd.

### Bioinformatics analysis

The raw sequencing data were subjected to initial quality filtering with Trimmomatic (version 0.33) to exclude low-quality sequences. The primer sequences were removed via Cutadapt (version 1.9.1) (Martin, 2011). USEARCH (version 10) was used to merge paired-end sequences (Edgar, 2013), and UCHIME (version 8.1) was used to remove chimeras (Edgar et al., 2011), ensuring high-quality sequences for further analysis. Denoising of quality-controlled data was performed using the DADA2 algorithm (Callahan et al., 2016) in QIIME2 (2020.6 version) (Bolyen et al., 2019), with a 0.005% threshold to filter anomalous sequences. Operational taxonomic units (OTUs) were clustered at a 97% sequence similarity threshold and taxonomically classified using the RDP classifier against the RDP Bacterial 16S rRNA (Wang et al., 2007) and UNITE Fungal ITS (Kõljalg et al., 2013) databases.

After quality control, 12,250 bacterial and 3,756 fungal OTUs were identified. The Chao 1 and Shannon indices were used to assess species richness and community diversity, respectively. Beta diversity was analyzed using principal coordinate analysis (PCoA), multiple regression tree (MRT; De'Ath, 2002), and heatmap clustering on the basis of the Bray–Curtis distance. Permutational multivariate analysis of variance (PERMANOVA) and analysis of similarity (ANOSIM) were used to test for differences in community structure. Hellinger standardization was performed on the rarefied OTU table before beta diversity was analyzed. The relative abundance of each OTU per sample was calculated as the number of sequences affiliated with the target OTU divided by the total number of sequences. Structural equation models (SEMs), constructed with the GGally, lavaan, and semPlot packages, were used to evaluate the influence of bacterial and fungal communities on pathogens (Mamet et al., 2019). In the structural equation model, soil microbial community structure (PCoA1 scores) and diversity (Shannon index) were included as measured variables, with the abundance of *F. oxysporum* as the response variable.

### Identification of the core microbiome

We first applied linear discriminant analysis effect size (LEfSe) with a threshold of scores ≥3 to identify differential microbial biomarkers across various spice crops (Hong et al., 2020). Subsequently, the predictive importance of these biomarkers in suppressing *F. oxysporum* was evaluated using a random forest model implemented through the R package randomForest (Wen et al., 2023b). We subsequently identified hub OTUs among the rotated crops. The psych package in R was used for graphical representation, and data points with correlation coefficients above 0.8 and *P* < 0.05 were selected to construct a bacterial–fungal co-occurrence network (Revelle and Revelle, 2015). The topological properties of this network were assessed using GEPHI software for visualization (Bastian et al., 2009). A high average degree of nodes indicated increased network complexity, implying a more intricate interplay among microbial species. Hub OTUs was identified on the basis of a customized threshold reflecting node characteristics: a degree > 7 and closeness centrality > 0.6, which accounted for <15% of all nodes in the network (Costello et al., 2012; Agler et al., 2016; Banerjee et al., 2018). Ultimately, the core microbiome was identified via the intersection of biomarker microbes identified through LEfSe and random forest analysis with hub microbes identified through network analysis (Hong et al., 2023).

### Statistical analysis

A comparison of pathogen abundance, microbial diversity, taxonomic composition, and soil physicochemical factors was conducted using Duncan's multiple range test in the agricolae package of R. We employed the vegan package in R to conduct both linear and nonlinear regression analyses, thereby investigating the relationship between pathogen abundance and microbial diversity. The rdacca.hp R package was employed to evaluate the relative importance of soil physicochemical factors as explanatory variables influencing bacterial and fungal microbial communities (Lai et al., 2022). Furthermore, we applied a general linear model (GLM) using the relaimpo package in R to evaluate the contributions of bacterial and fungal communities, core microbes, and physicochemical factors to pathogen suppression (Shen et al., 2019).

### Sequence accession numbers

The sequences have been submitted to the Sequence Read Archive (SRA) of the National Center for Biotechnology Information under the accession number PRJNA1126394.

## Results

### Disease incidence, pathogen abundance, and rhizosphere microbial structure

**Figure 1:**
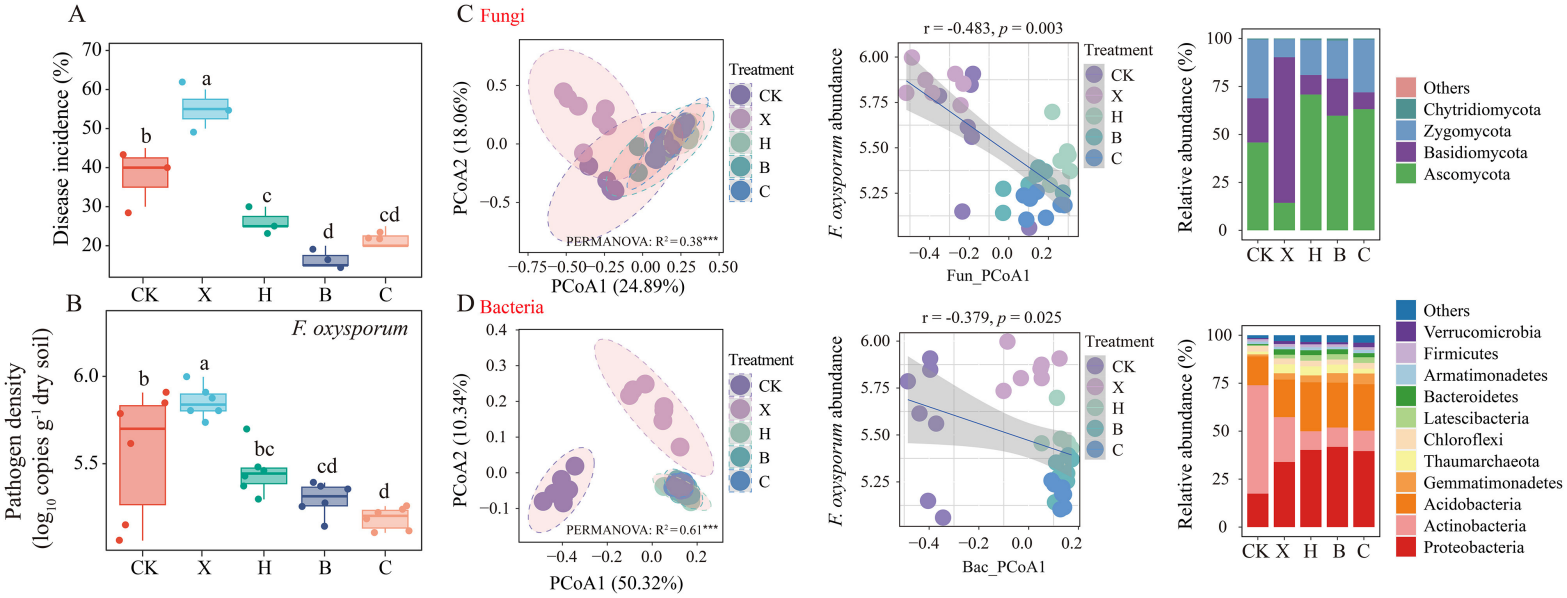
Impact of crop rotation on disease incidence and *Fusarium oxysporum* abundance in vanilla rhizosphere soil. Effects of crop rotation on **(A)** disease incidence and **(B)** *Fusarium oxysporum* abundance. Changes in rhizosphere soil **(C)** fungal and **(D)** bacterial community structure and composition across crops. Linear regression analysis of community structure and *F. oxysporum* abundance. Statistical significance was assessed via Duncan's test (mean ± SD). Different letters denote a significant difference at the *P* < 0.05 level. CK, fallow; X, vanilla monoculture; H, black pepper–vanilla rotation; B, pandan–vanilla rotation; C, sweet rice tea–vanilla rotation.

After a decade of continuous vanilla cultivation, a rotation with black pepper, pandan, and sweet rice tea was performed before replanting with vanilla. We found that the DI of vanilla was 55% with continuous cultivation, but it significantly decreased to 27% after rotation with black pepper, 22% with sweet rice tea, and 17% with pandan (Figure 1A). *F. oxysporum* abundance was significantly lower in the pandan–vanilla and sweet rice tea–vanilla rotation systems (*P* < 0.05), with no significant difference between the two treatments (Figure 1B).

Compared with the vanilla monoculture, crop rotation with black pepper, sweet rice tea, or pandan did not significantly affect soil bacterial richness or diversity, but both richness and diversity were significantly greater in the rotations than those in the fallow treatment (Supplementary Figures S1A, B). The richness and diversity of the fungal community significantly increased with rotation (Supplementary Figures S1C, D) and were significantly negatively correlated with pathogen abundance (Supplementary Figures S1G, H). Principal coordinate analysis (PCoA) based on Bray–Curtis distances clearly revealed significant differences in the soil fungal communities among the fallow, vanilla monoculture, and crop rotation treatments (Figure 1C, left). However, no distinct separation was observed among the rotations of black pepper, pandan, and sweet rice tea. This finding was confirmed by PERMANOVA (Figure 1C, *R*^2^ = 0.38, *P* = 0.001) and ANOSIM (Supplementary Table S1, *P* = 0.001). Moreover, the fungal community (PCoA1) was significantly negatively correlated with the abundance of *F. oxysporum* (Figure 1C, middle). Further cluster heatmap analysis indicated that fallow and vanilla monoculture could be clustered separately, whereas the rotations of pepper, pandan and sweet rice tea had similar community structures and were clustered together (Supplementary Figure S2B). The trend in the soil bacterial community structure was consistent with that of the fungal community structure (Figure 1D).

ITS sequencing revealed that, compared with vanilla monoculture, crop rotation with pepper, pandan and sweet rice tea significantly increased the relative abundance of the phylum *Ascomycota* (Figure 1C, right) and was significantly negatively correlated with *F. oxysporum* abundance (Supplementary Table S2). The genera *Thermomyces* and *Arthrobotrys* were significantly more abundant after rotation, with *Arthrobotrys* increasing 9.8-fold after pandan rotation, and both genera were negatively correlated with the pathogen (Supplementary Table S3). 16S rRNA gene sequencing revealed that crop rotation significantly increased the abundance of *Proteobacteria, Gemmatimonadetes*, and *Latescibacteria* (Figure 1D, right), which was also negatively correlated with *F. oxysporum* abundance (Supplementary Table S2). At the genus level, *Nitrosospira, Gemmatimonas*, and *Gp17* were significantly more abundant in rotation systems, particularly those involving pandan and sweet rice tea, and were negatively associated with the pathogen (Supplementary Table S4).

### Disease suppression by rhizosphere microbial communities

**Figure 2:**
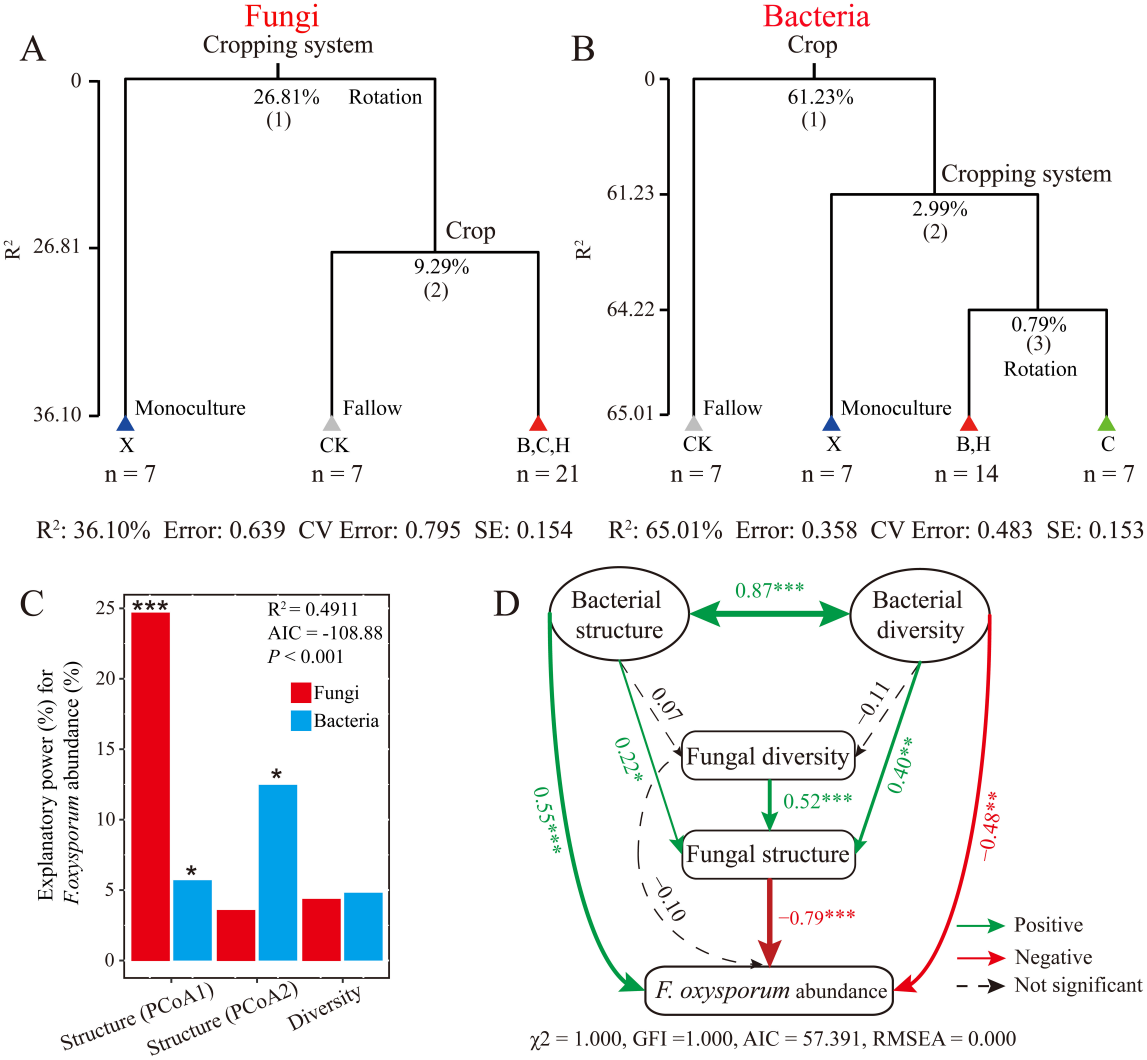
Analyses of soil microbial community structure and pathogen inhibitory effects. Multiple regression tree (MRT) analysis of soil microbial **(A)** fungal and **(B)** bacterial community structure; **(C)** relative importance of community structure in pathogen inhibition; **(D)** structural equation modeling (SEM) analysis of pathogen inhibition correlations, with solid arrows indicating significant (*P* < 0.05) and dashed arrows indicating non-significant correlations. The R-squared values indicate the correlation strength and direction. Positive values indicate positive correlations, and negative values indicate negative correlations. CK, fallow; X, vanilla monoculture; H, black pepper–vanilla rotation; B, pandan–vanilla rotation; C, sweet rice tea–vanilla rotation.

MRT analysis revealed that cropping system was the main factor shaping rhizosphere fungi, explaining 26.81% of the variance, whereas crops in rotation were secondary factors, accounting for 9.29% of the variance (Figure 2A). For bacteria, crop rotation was the primary factor, explaining 61.23% of the variance (Figure 2B).

A general linear model (GLM) was used to assess the impact of fungal and bacterial community structure and diversity on the suppression of *F. oxysporum* abundance, quantified via qPCR-based absolute quantification (Figure 2C). Both the fungal and bacterial principal coordinate axes significantly reduced pathogen abundance, with the fungal community being the major factor, accounting for 24.95% of the variance. The second axis of the bacterial community also significantly contributed, accounting for 13% of the variance. SEM revealed that bacterial community structure and diversity positively influence fungal community structure (*r* = 0.22 for structure, *r* = 0.40 for diversity), which in turn significantly decreased the abundance of *F. oxysporum* quantified via qPCR (*r* = −0.79; Figure 2D).

### Identification of disease-suppressive biomarkers

**Figure 3:**
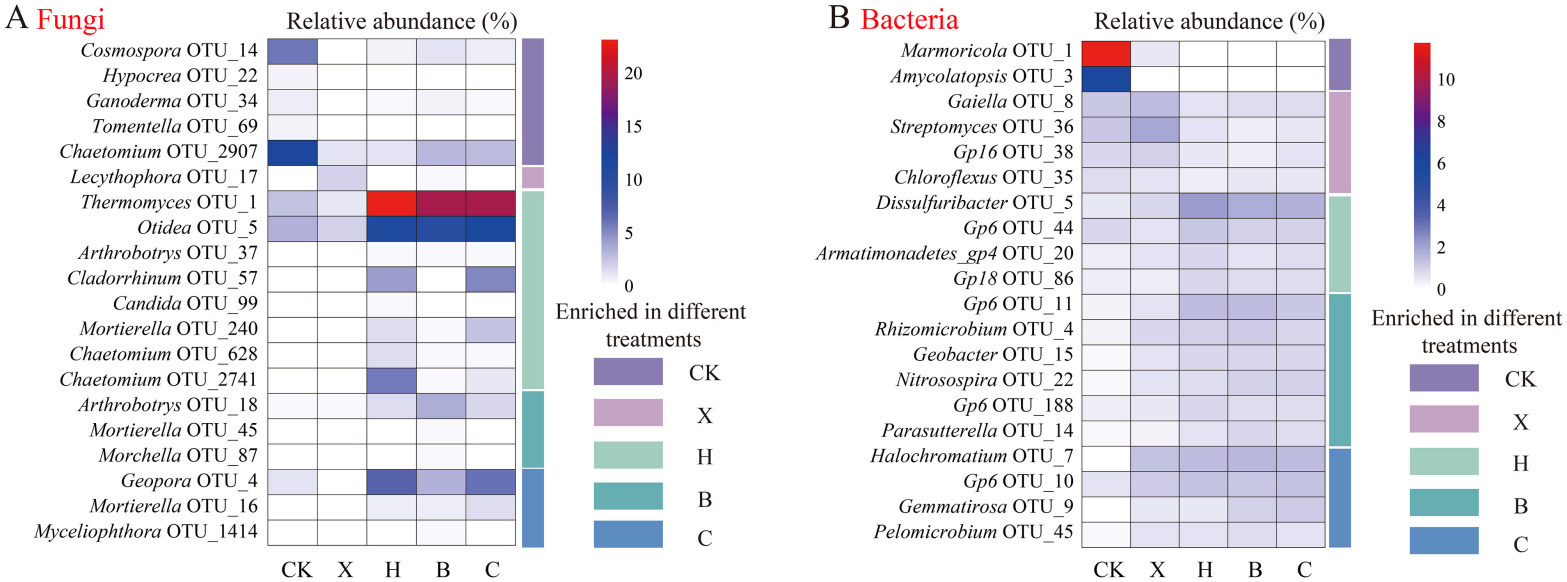
Relative abundance of biomarkers of rhizosphere microbiomes in different spice crops identified by linear discriminant analysis (LDA). Identification of **(A)** fungal and **(B)** bacterial biomarkers via LDA. Different lowercase letters denote significance at *P* < 0.05. CK, fallow; X, vanilla monoculture; H, black pepper–vanilla rotation; B, pandan–vanilla rotation; C, sweet rice tea–vanilla rotation.

We used a more stringent LDA score threshold of ≥3 to identify biomarkers between groups. We then displayed the top 20 fungal (Figure 3A) and bacterial (Figure 3B) OTU taxa by relative abundance. Among fungi, 20 biomarkers were identified (Supplementary Table S5). Specifically, the fallow treatment enriched 5 biomarkers, mainly from the phyla *Ascomycota* and *Basidiomycota*; vanilla monoculture enriched 1 *Ascomycota* biomarker; black pepper rotation enriched 8 biomarkers, primarily from the phyla *Ascomycota* and *Zygomycota*; pandan rotation enriched 3 *Zygomycota* biomarkers; and sweet rice tea rotation enriched 3 biomarkers from the phyla *Ascomycota* and *Zygomycota*. Notably, black pepper rotation significantly enriched OTU1_*Thermomyces* (F = 4.682, *P* = 0.005) and OTU37_*Arthrobotrys* (F = 4.188, *P* = 0.008), pandan rotation significantly enriched OTU18_*Arthrobotrys* (F = 3.326, *P* = 0.023), and sweet rice tea rotation significantly enriched OTU16_*Mortierella* (F = 2.800, *P* = 0.044). The relative abundances of these biomarkers were significantly negatively correlated with *F. oxysporum* abundance (*P* < 0.05).

For bacteria, we identified a total of 147 biomarkers (Supplementary Table S6). Specifically, the fallow treatment enriched 27 biomarkers, mainly from the phyla *Proteobacteria* and *Actinobacteria*; vanilla monoculture enriched 22 biomarkers, primarily from the phyla *Proteobacteria* and *Actinobacteria*; black pepper rotation enriched 26 biomarkers, mainly from the phyla *Proteobacteria* and *Acidobacteria*; pandan rotation enriched 34 biomarkers, primarily from the phyla *Proteobacteria* and *Acidobacteria*; and sweet rice tea rotation enriched 38 biomarkers, predominantly from the phylum *Proteobacteria*.

**Figure 4:**
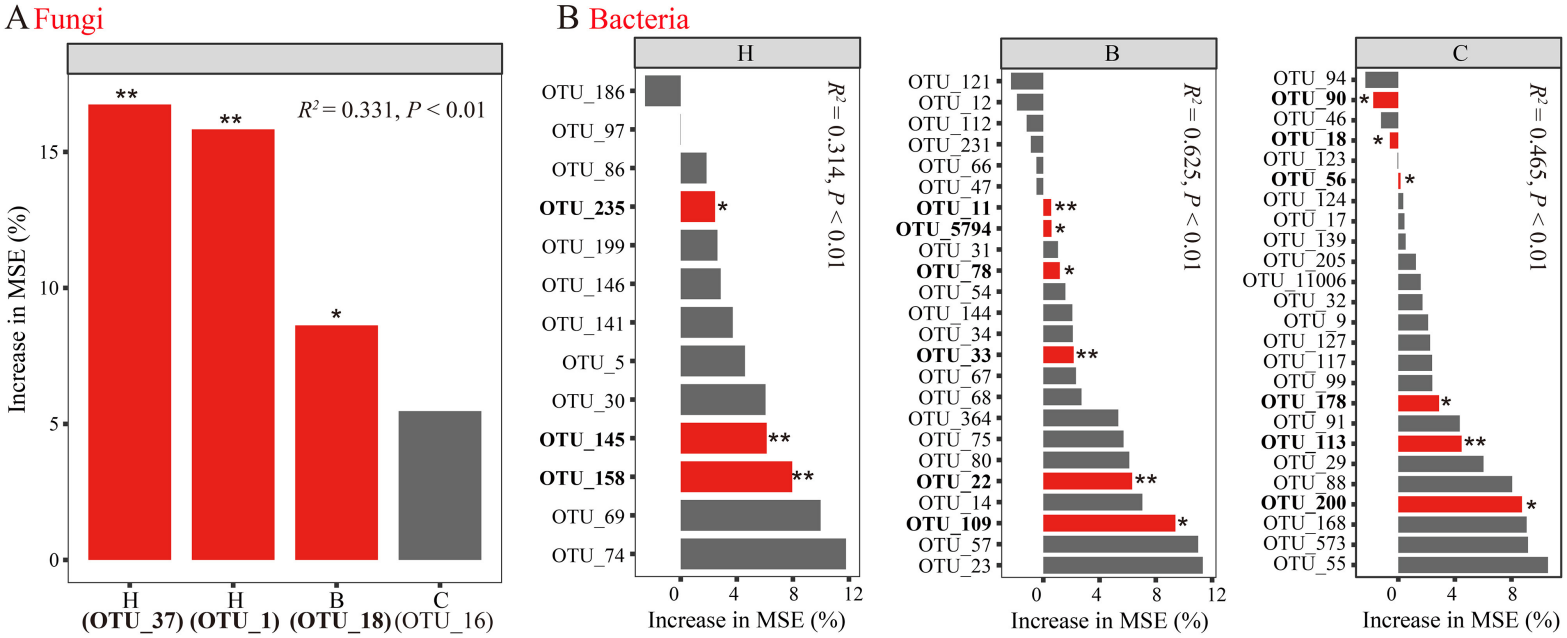
Assessment of **(A)** fungal and **(B)** bacterial biomarker responses to *F. oxysporum* with random forest. Different lowercase letters denote significance at *P* < 0.05. CK, fallow; X, vanilla monoculture; H, black pepper–vanilla rotation; B, pandan–vanilla rotation; C, sweet rice tea–vanilla rotation.

Random forest models identified significant predictors of *F. oxysporum* response. In the fungal model (Figure 4A, *R*^2^ = 0.331, *P* < 0.01), black pepper–vanilla rotation enriched OTU1_*Thermomyces* and OTU37_*Arthrobotrys*, and pandan–vanilla rotation enriched OTU18_*Arthrobotrys* significantly. For bacteria (Figure 4B), the black pepper–vanilla rotation model (*R*^2^ = 0.314, *P* < 0.01) highlighted OTU235_*Gp16*, OTU145_*Gp11*, and OTU158_*Gp17* as predictors. In the pandan–vanilla rotation model (R^2^ = 0.625, *P* < 0.01), multiple bacterial OTUs were identified as predictors, including OTU11_*Gp6*, OTU5794_*Magnetospirillum*, OTU78_*Zoogloea*, OTU33_*Lacibacterium*, OTU22_*Nitrosospira*, and OTU109_*Latescibacteria*. The sweet rice tea–vanilla rotation model (*R*^2^ = 0.465, *P* < 0.01) also revealed several significant bacterial predictors, such as OTU200_*Methylibium*, OTU113_*Roseisolibacter*, OTU178_*Actinospica*, OTU56_*Lacibacterium*, OTU18_*Nitrospira*, and OTU90_*Micromonospora*. Compared with those in the fallow and vanilla monoculture treatment, the relative abundances of the identified biomarkers significantly increased (Supplementary Figure S3, *P* < 0.05).

### Identification of hub taxa

**Figure 5:**
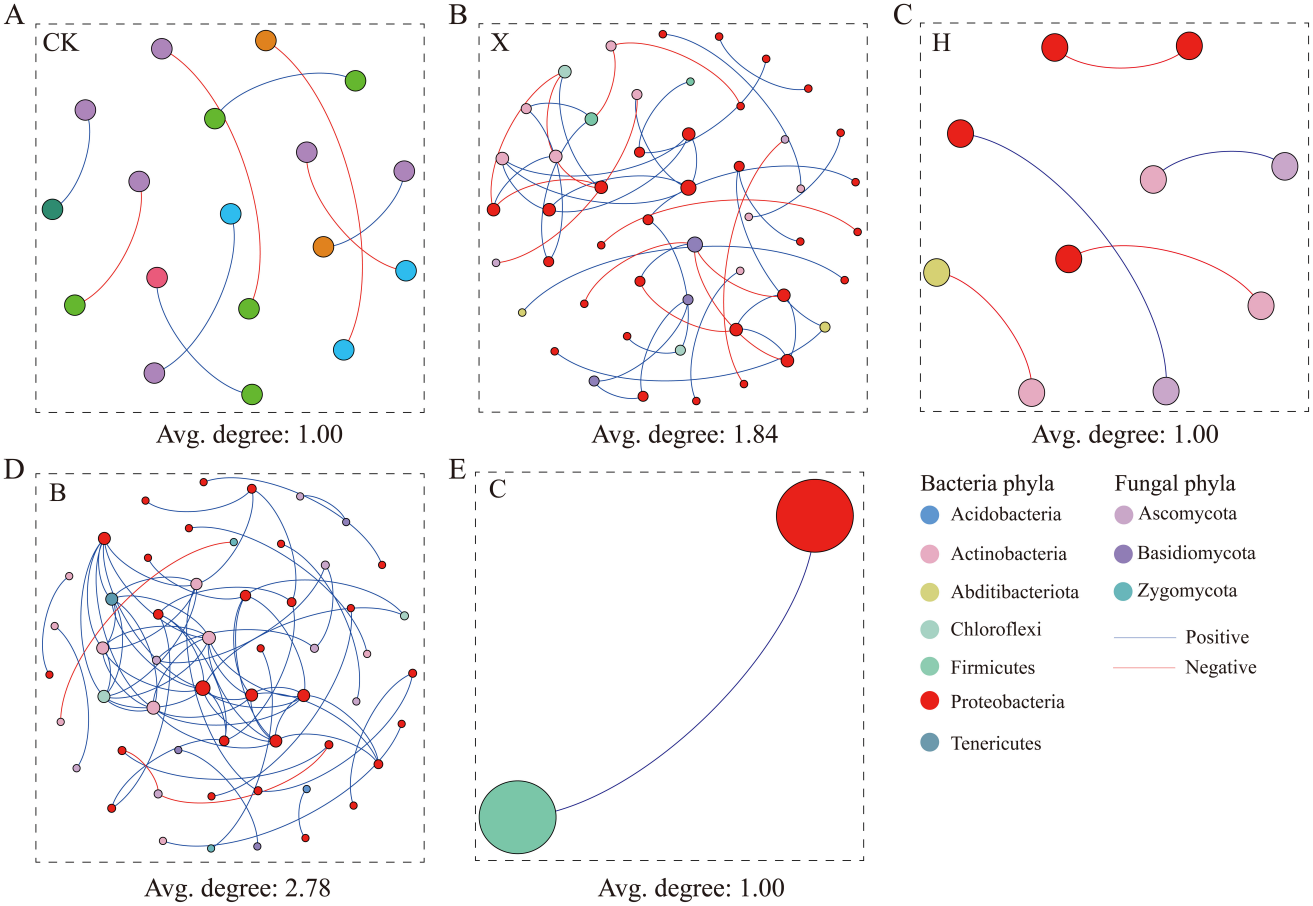
Assessing microbial co-occurrence networks in the rhizospheres of rotated spice crops. Complexity of bacterial–fungal interactions reflected by network node connectivity. A higher average degree of nodes indicates a more complex network, suggesting more extensive synergistic interactions between microbiomes. **(A)** CK = fallow; **(B)** X = vanilla monoculture; **(C)** H = black pepper-vanilla rotation; **(D)** B = pandan-vanilla rotation; **(E)** C = sweet rice tea-vanilla rotation.

Co-occurrence network analysis of rhizosphere bacteria and fungi revealed distinct structures under fallow, vanilla monoculture, and rotation treatments (Figure 5). The pandan–vanilla rotation network had the highest node and connection counts, with 96% positive correlations, and the highest average node degree was 2.78, indicating strong microbial interactions (Supplementary Table S7). On the basis of node degree and closeness centrality (Table 1), in the pandan–vanilla rotation, 8 bacterial hub taxa, such as OTU31, OTU99, OTU178, OTU10, OTU14, OTU22, OTU56, and OTU130, were uniquely identified. The 8 identified hub taxa had relative abundances >0.2%, with values of 0.53%, 0.27%, 0.28%, 1.26%, 0.87%, 1.01%, 0.27%, and 0.38%, respectively. Notably, OTU22, OTU56, and OTU178, identified as enriched biomarkers by random forest analysis, also qualified as hubs in the network analysis, designating them as core microbiota. OTU22, specifically, emerged as a pandan–vanilla rotation-enriched, host-specific core microbe.

**Table 1:** Identification of hub nodes in bacterial–fungal microbial co-occurrence networks after rotation with various spice crops.

**Network**	**Sample ID^b^**	**Phylum**	**Genus**	**RA%^c^**	**Degree^d^**	**Closeness^e^ centrality**
B_network^**a**^	B_OTU31	*Acidobacteria*	*Gp6*	0.53%	10	0.6
	B_OTU99	*Nitrospirae*	*Nitrospira*	0.27%	8	0.6
	B_OTU178	*Actinobacteria*	*Actinospica*	0.28%	8	0.6
	B_OTU10	*Acidobacteria*	*Gp6*	1.26%	7	0.6
	B_OTU14	*Proteobacteria*	*Parasutterella*	0.87%	7	0.6
	B_OTU22	*Proteobacteria*	*Nitrosospira*	1.01%	7	0.6
	B_OTU56	*Tenericutes*	*Lacibacterium*	0.27%	7	0.6
	B_OTU130	*Acidobacteria*	*Gp6*	0.38%	7	0.6

^a^ The B_network refers to the bacterial-fungal microbial co-occurrence network in the rhizosphere soil of pandan. According to our network node characteristic criteria for identifying hub nodes (i.e., degree and closeness centrality), the rhizosphere microbial co-occurrence networks of other spice crops exhibited no hub nodes meeting these established standards.

^b^ B_OTUs denote bacterial taxa.

^c^ RA (relative abundance) refers to the percentage representation of each bacterial taxon within the microbial community of pandan.

^d/e^ Hub OTUs were identified based on a customized threshold reflecting node characteristics: a degree > 7 and a closeness centrality > 0.6. These hub OTUs accounted for <15% of all nodes in the network.

### Predicting pathogen-suppressive indicators

**Figure 6:**
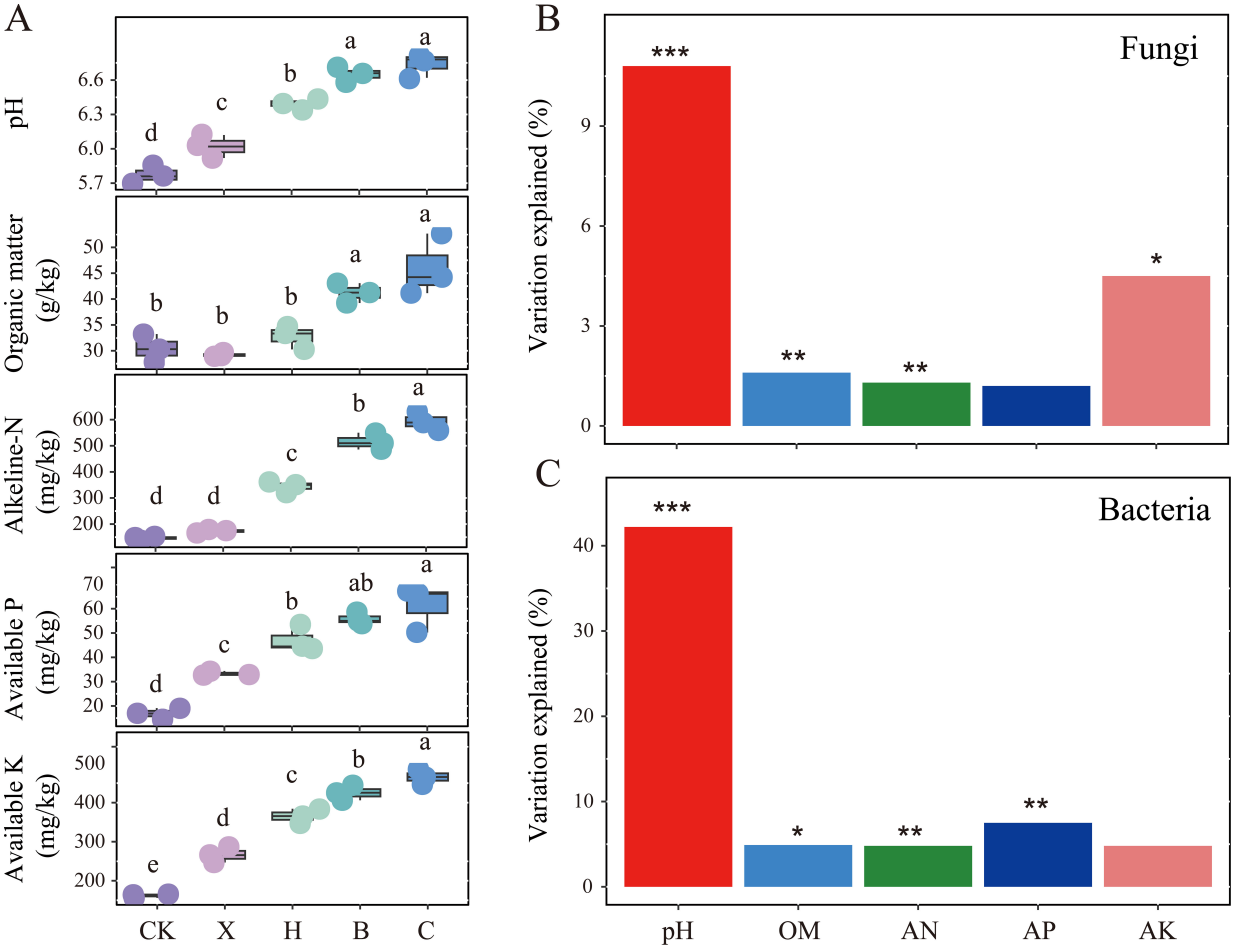
Variations in **(A)** soil physicochemical properties and the impact of these environmental factors on the composition of **(B)** fungal and **(C)** bacterial communities after rotation with different spice crops. CK, fallow; X, vanilla monoculture; H, black pepper–vanilla rotation; B, pandan–vanilla rotation; C, sweet rice tea–vanilla rotation. OM, organic matter; NA, alkali-hydrolysable, AP, available phosphorus; AK, available potassium. * represents *P* < 0.05, ** represents *P* < 0.01 and *** represents *P* < 0.001.

Following crop rotation with spice crops, we measured soil physicochemical properties and found that, compared with those under vanilla monoculture cultivation, soil pH, OM, AN, AP, and AK significantly increased under spice rotation (Figure 6A, *P* < 0.05). However, soil pH and OM did not differ significantly between the pandan and sweet rice tea rotation systems. Soil pH and OM significantly influenced both fungal and bacterial communities, with soil pH being the primary factor, explaining 10.8% and 42.2% of the variance in fungal and bacterial communities, respectively (Figures 6B, C).

A general linear model was used to evaluate the potential of microbial communities, core microbiota and physicochemical properties in suppressing pathogens. The model explained 71.02% of the variation in pathogen suppression (Table 2, *P* < 0.001). Key factors included fungal community structure, the core microbial species OTU22, and soil pH, with individual explanatory contributions of 18.71%, 7.59%, and 17.83%, respectively (*P* < 0.05 for all).

**Table 2:** Linear modeling to identify the microbial and soil physicochemical factors associated with the reduction in *Fusarium oxysporum* abundance in vanilla.

***F. oxysporum* abundance**	**df**	***F* value**	***P*-value**	***t***	**RI (%)**
**Fungal structure**	**1**	**42.373**	**0.001**	**−1.939**	**18.71%**
Bacterial structure	1	0.240	0.628	3.259	8.48%
OTU_18 (*Arthrobotrys*)	1	1.023	0.321	0.244	2.45%
OTU_37 (*Arthrobotrys*)	1	0.593	0.447	**–**1.058	6.88%
OTU_11 (*Gp6*)	1	0.402	0.531	**–**0.345	3.97%
**OTU_22 (** ***Nitrosospira*** **)**	**1**	**10.835**	**0.002**	**−2.096**	**7.59%**
OTU_56 (*Lacibacterium*)	1	0.476	0.496	**–**0.310	2.48%
OTU_178 (*Actinospica*)	1	0.197	0.663	0.504	2.63%
**pH**	**1**	**5.132**	**0.032**	**−2.265**	**17.83%**
Residuals	27				
**Model summary:** **R^2^** **=** **0.7102, AIC** **=** **−116.23**, ***p*** **<** **0.001**					
Proportion of variance explained by model: 71.02%					

RI, relative importance. *P*-values were calculated via ANOVA. Bolded lines in the table represent factors exhibiting a statistically significant correlation with decreased pathogen abundance (*P* < 0.05).

## Discussion

Vanilla, an herbaceous perennial vine, is particularly susceptible to continuous cropping obstacles. Our previous research revealed that rotation with black pepper can effectively regulate the soil fungal community, thereby increasing vanilla disease resistance (Xiong et al., 2016). In this study, we conducted pot experiments by replanting vanilla in soils previously rotated with black pepper, pandan and sweet rice tea. The results indicated that rotation with pepper, pandan, or sweet rice tea not only reduced the DI but also significantly decreased the copy number of *F. oxysporum* (Figure 1). This aligns with our previous findings on crop rotation systems. For example, cultivation methods such as pepper–banana rotation (Hong et al., 2020), eggplant–banana rotation (Hong et al., 2023) and pineapple–banana rotation (Yuan et al., 2021) effectively improved banana disease suppression. These findings suggest that reducing the abundance of *F. oxysporum* may be key to enhancing vanilla disease suppression. However, owing to the high cost of planting and managing vanilla, we opted for pot experiments over field trials. Although the experiment was conducted in a controlled environment, we adjusted parameters such as temperature and humidity to mimic natural microenvironments, ensuring the reliability, stability and reproducibility of our results. In addition, pandan and sweet rice tea are short-cycle spice crops that have been widely applied in tropical production in recent years. The production cycle of these plants is 6 months, whereas that of black pepper is 30 months. Therefore, considering the planting cycles of black pepper, pandan and sweet rice tea, we selected a planting time that was in between these two cycles for our pot experiment. Specifically, we replanted vanilla in the soil after a 12-month rotation to evaluate disease-suppression effects. Although our current study has certain limitations, we plan to conduct further long-term research to explore the differences in disease-suppression effects across various rotation cycles.

The diversity and richness of the soil microbiome are pivotal for alleviating the negative impacts of environmental stress on plants, offering protection against biotic challenges and assisting in the plant recovery process (Trivedi et al., 2020). Our study revealed that the adoption of a crop rotation regime that included black pepper, pandan, or sweet rice tea markedly improved the richness and diversity of the soil fungal community. This finding corroborates the outcomes of our previous research, indicating a pronounced increase in the richness and diversity of fungal populations when black pepper and coffee were under rotation with vanilla (Xiong et al., 2016). These results highlight the diverse impacts of different crop rotation strategies on the bacterial and fungal constituents of the soil microbiome. Specific rotational crops appear to stimulate the proliferation of beneficial microbial groups, thereby shaping the structure of the soil microbial community. These microbes compete with pathogens for niche resources at the root interface, limiting pathogen access to nutrients and effectively controlling the spread of the pathogens (Wen et al., 2023a).

Studies indicate that the structure and composition of microbial communities after long-term crop rotation significantly differ from those in monoculture systems (Zhou et al., 2023; Cerecetto et al., 2024). In this study, both fungal and bacterial community structures were clearly distinguishable among fallow, monoculture and crop rotation treatments, resulting in three distinct community configurations. However, the community structures of the black pepper, pandan and sweet rice tea groups were notably similar and indistinguishable. This similarity suggests that crop rotation alters soil resource distribution and environmental conditions, allowing different functional microbial communities to coexist simultaneously, leading to overlapping communities. Essentially, the soil microbial communities that establish following rotation with black pepper, pandan and sweet rice tea likely contain beneficial microbes that compete with pathogens for limited rhizosphere resources. Therefore, future research should be focused on elucidating the mechanisms of community assembly to better understand how crop rotation affects the composition of soil microbial communities, thereby enhancing crop health.

In this study, we conducted a comparative analysis of microbes identified as biomarkers by LDA and random forest analysis and identified hub taxa from co-occurrence networks, defining core microbes as those common to both. We discovered that replanting vanilla in soils previously subjected to rotation with black pepper, pandan, and sweet rice tea specifically activated certain fungal biomarker taxa, such as OTU1_*Thermomyces*, OTU18_*Arthrobotrys*, and OTU37_*Arthrobotrys*, as well as core bacterial microbes, such as OTU22_*Nitrosospira*, OTU56_*Lacibacterium*, and OTU178_*Actinospica*. Our previous research indicated that black pepper–vanilla rotation could significantly enrich antagonistic core fungal taxa, such as *Trichoderma* and *Penicillium* (Xiong et al., 2016). This implies that different rotation crops possess distinct rhizosphere metabolites, which, during the vanilla season, differentially enrich specific core microbiota (Wen et al., 2023b). Additionally, the duration of crop rotation significantly influences the assembly of core microbial communities (Iheshiulo et al., 2023). Existing studies have demonstrated that fungal taxa, such as *Thermomyces* and *Arthrobotrys*, may contribute positively to disease suppression (Nagai et al., 2020), but there is currently insufficient evidence to assert that core bacterial microbes, such as *Nitrosospira, Lacibacterium*, and *Actinospica*, have direct disease resistance functions. Nevertheless, the core microbes identified in this study are integral components of the soil microbial community (Yue et al., 2024). Different members of these core groups may suppress diseases through various mechanisms, including antibiotic production (Li et al., 2016; Kusuma et al., 2022; Banerjee and van der Heijden, 2023), nutrient competition, or physical barrier formation. Moreover, these core taxa may indirectly influence pathogen growth through synergistic interactions with other microbes (Li et al., 2016; Banerjee and van der Heijden, 2023).

Research has shown that crop rotation, for example, introducing chili peppers in banana plantations, can restructure the soil microbiome by increasing pH, leading to increased proliferation of beneficial *Pseudomonas* that improve disease resistance in crops (Hong et al., 2020). Our study corroborates these findings, demonstrating that intercropping vanilla with spice crops significantly increases rhizosphere soil pH and impacts fungal and bacterial communities. Crop rotation effectively mitigates the acidification trend induced by monoculture practices through mechanisms including (1) modulating the chemical properties of crop residues, (2) optimizing nutrient uptake balance, (3) reshaping microbial functional profiles, and (4) enhancing soil buffering capacity. The pronounced sensitivity of soil microbial communities to pH variations indicates that agricultural practices can be leveraged to increase crop disease resistance (Tamburini et al., 2020; Wittwer et al., 2021). For example, amending the soil pH with peanut crop residues can increase the activity of the antagonistic soil microbiome (Watanabe et al., 2011). The pH level directly influences the enzymatic activity and physiological processes within *Fusarium*, thereby regulating their growth and metabolism. Additionally, pH significantly and directly affects the toxin-producing capacity of *Fusarium*. pH modulates the composition of plant root exudates (e.g., organic acids and phenolic compounds), indirectly altering the host plant's chemical defense efficacy against *Fusarium*. Furthermore, increasing the soil pH can regulate the soil microbial community by enriching beneficial bacteria and suppressing pathogens, thereby alleviating soil-borne diseases in *Panax notoginseng* (Deng et al., 2024). Through a general linear model, we determined that fungal community structure, particularly the core taxon OTU2, and soil pH are key determinants of pathogen suppression, with fungal structure being the predominant factor. This underscores the need to consider the collective influence of soil microbial communities on the health of crops rather than focusing solely on individual microbiota (Wagg et al., 2019; Delgado-Baquerizo et al., 2021).

In summary, intercropping spice crops, particularly pandan, with orchards susceptible to soil-borne diseases effectively reduces the incidence of disease and the level of *F. oxysporum* while increasing fungal community diversity. This approach selectively enriches for key fungal and bacterial taxa, such as OTU1*_Thermomyces*, OTU18_*Arthrobotrys*, OTU37_*Arthrobotrys*, OTU22_*Nitrosospira*, OTU56_*Lacibacterium*, and OTU178_*Actinospica*, reshaping the soil microbiome. The resulting increase in soil pH is believed to reorganize microbial communities, particularly fungal communities, thereby suppressing pathogen abundance. These results suggest that the modulation of soil pH and restructuring of microbial communities are pivotal for enhancing vanilla resistance to diseases through the pandan–vanilla rotation strategy.

## Data Availability

The datasets presented in this study can be found in online repositories. The names of the repository/repositories and accession number(s) can be found in the article/Supplementary material.
