# Supplementary material for: Pandan–vanilla rotation mitigates Fusarium wilt disease in vanilla: insights from rhizosphere microbial community shifts
Source: Front Microbiol. 2025 Jun 18;16:1496701. doi: 10.3389/fmicb.2025.1496701 (PMC12213491; doi:10.3389/fmicb.2025.1496701)
Supplement: Supplementary file 1 [file Data_Sheet_1.pdf]

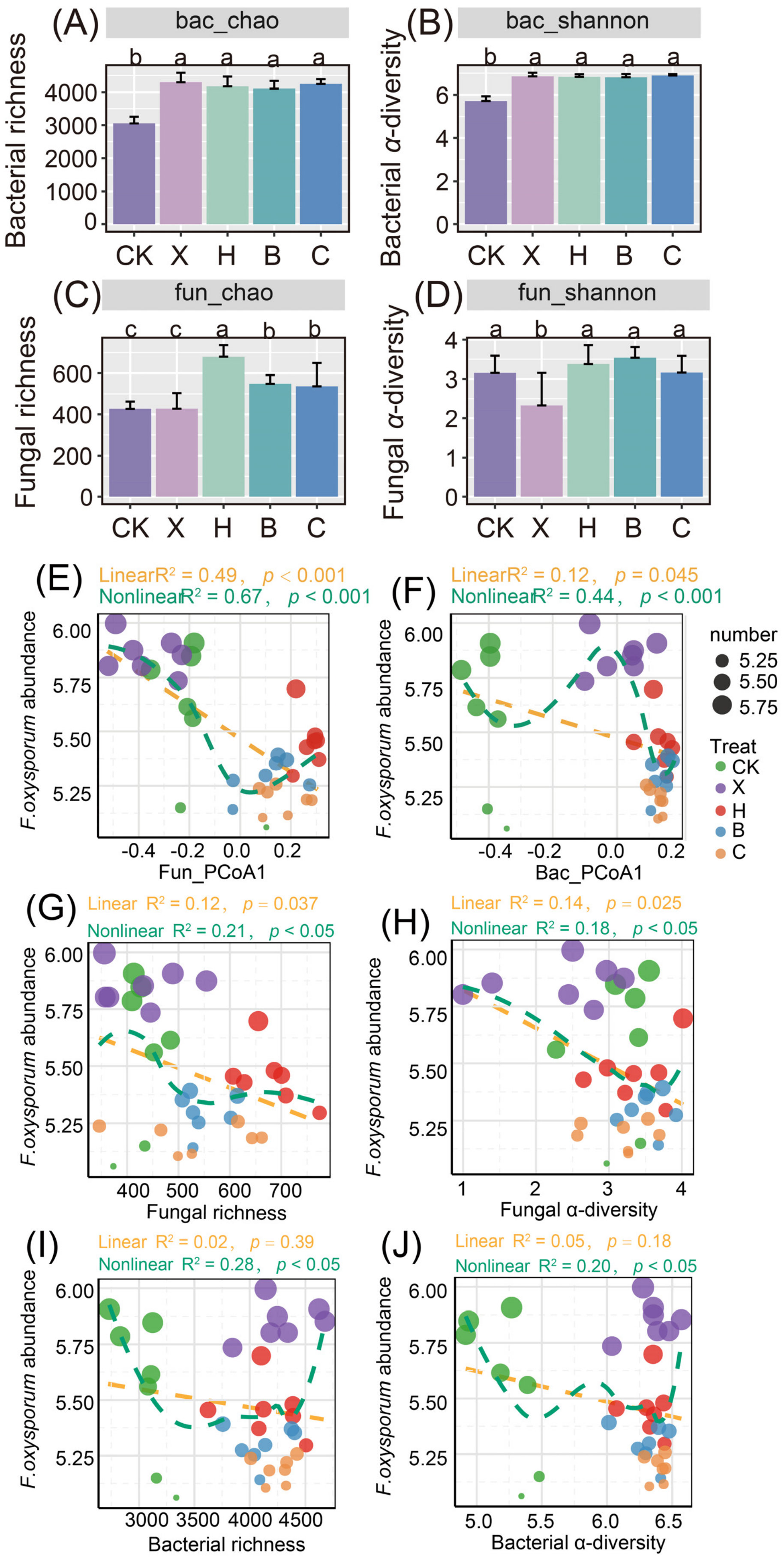

(A) bac_chao
Bacterial richness
CK X H B C
(B) bac_shannon
Bacterial α-diversity
(C) fun_chao
Fungal richness
(D) fun_shannon
Fungal α-diversity
(E) LinearR² = 0.49, p < 0.001
NonlinearR² = 0.67, p < 0.001
F.oxysporum abundance
Fun_PCoA1
(F) LinearR² = 0.12, p = 0.045
NonlinearR² = 0.44, p < 0.001
Bac_PCoA1
number
5.25
5.50
5.75
Treat
CK
X
H
B
C
(G) Linear R² = 0.12, p = 0.037
Nonlinear R² = 0.21, p < 0.05
Fungal richness
(H) Linear R² = 0.14, p = 0.025
Nonlinear R² = 0.18, p < 0.05
Fungal α-diversity
(I) Linear R² = 0.02, p = 0.39
Nonlinear R² = 0.28, p < 0.05
Bacterial richness
(J) Linear R² = 0.05, p = 0.18
Nonlinear R² = 0.20, p < 0.05
Bacterial α-diversity

Figure S1 Comparing the abundance and diversity of bacterial and fungal communities and their correlation with the pathogen using both linear and nonlinear regression analyses. Different letters denote a significant difference at the $P < 0.05$ level. CK = fallow; X = vanilla monoculture; H = black pepper-vanilla rotation; B = pandan-vanilla rotation; C = sweet rice tea-vanilla rotation.

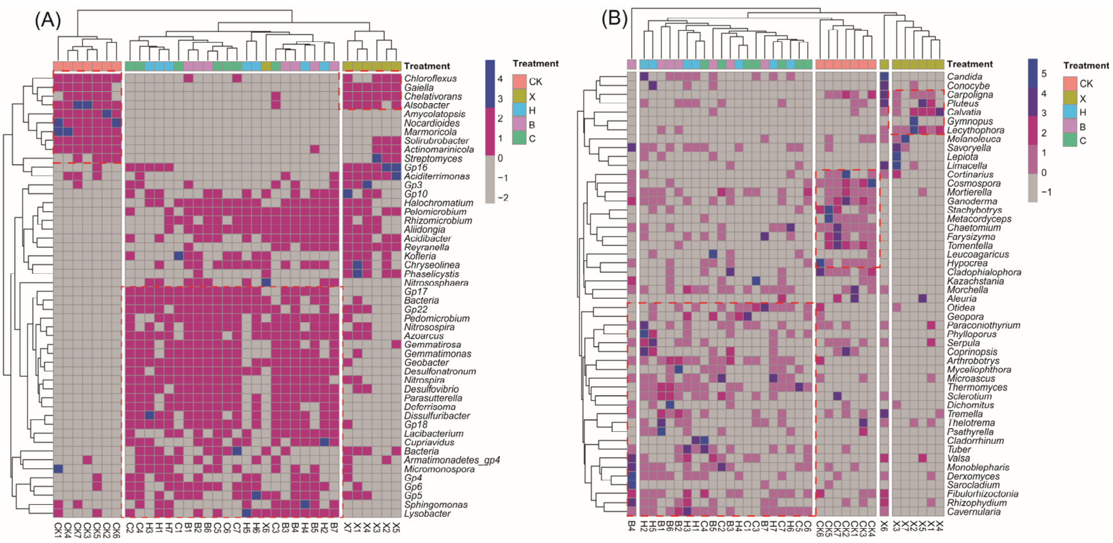

Figure S2 Heatmap analysis of the top 50 **(A)** bacterial and **(B)** fungal taxonomic groups clustered at the genus level. CK = fallow; X = vanilla monoculture; H = black pepper-vanilla rotation; B = pandan-vanilla rotation; C = sweet rice tea-vanilla rotation.

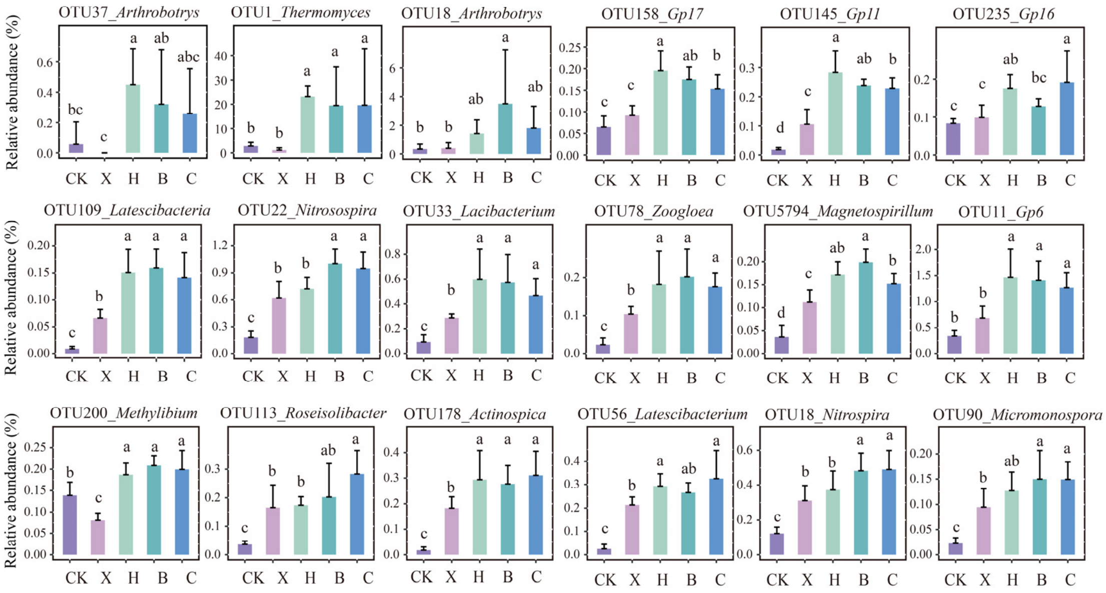

Figure S3 Significance testing of biomarkers via Duncan's Multiple Range Test. Different lowercase letters denote significance at $P < 0.05$. CK = fallow; X = vanilla monoculture; H = black pepper-vanilla rotation; B = pandan-vanilla rotation; C = sweet rice tea-vanilla rotation.

Table S1 PERMANOVA and ANOSIM results of bacterial and fungal community composition at the OTU level

	**Main test**	**PERMANOVA**			**ANOSIM**	
	**Factors**	**F**	R^2	***P*-value**	**R**	***P*-value**
Bacteria	Rotation	14.127***	0.247	0.001	0.733***	0.001
	variety	11.153***	0.195	0.001	0.935***	0.001
Fungi	Rotation	6.697***	0.152	0.001	0.636***	0.001
	variety	5.421***	0.123	0.001	0.818***	0.001

Table S2 Comparative analysis of bacterial and fungal phyla and Pearson and Spearman correlation analyses with vanilla pathogens

**Taxon**	**Phyla level**	**CK**	**X**	**H**	**B**	**C**	**Pearson correlation with *F. oxysporum* abundance**	**Spearman correlation with *F. oxysporum* abundance**
Bacterial	**Proteobacteria**	17.44±2.38c	33.95±5.55b	40.24±3.48a	41.83±2.19a	39.64±3.64a	-0.380*	-0.419*
	Actinobacteria	56.48±4.57a	23.35±11.86b	9.72±2.36c	10.06±2.42c	10.63±1.25c	0.348*	0.317
	Acidobacteria	15.07±3.01c	19.64±2.02b	25.49±3.41a	23.41±3.13a	24.23±2.48a	-0.328	-0.342*
	**Gemmatimonadetes**	1.05±0.13d	3.24±0.68c	3.62±0.51c	4.91±0.43b	5.58±0.85a	-0.486**	-0.517**
	Thaumarchaeota	1.46±0.90a	4.65±5.61a	4.71±2.28a	4.50±2.82a	2.47±0.76a	0.101	-0.055
	Chloroflexi	3.00±0.48a	3.09±0.40a	2.96±0.67a	2.64±0.23a	3.02±0.31a	0.033	0.009
	**Latescibacteria**	0.23±0.08c	1.94±0.36b	2.90±0.79a	2.77±0.58a	2.96±0.81a	-0.374*	-0.445**
	Bacteroidetes	0.64±0.22b	2.82±1.00a	2.56±0.64a	2.57±0.36a	2.16±0.40a	-0.033	-0.015
	Armatimonadetes	1.33±0.19ab	1.29±0.37b	1.53±0.37ab	1.23±0.18b	1.69±0.44a	-0.144	-0.114
	Firmicutes	1.20±0.17b	1.45±0.49ab	1.54±0.19a	1.34±0.20ab	1.41±0.18ab	0.056	-0.078
	Verrucomicrobia	0.59±0.19c	1.55±0.62ab	1.28±0.75bc	1.06±0.74bc	2.38±1.25a	-0.078	-0.012
Fungal	**Ascomycota**	45.79±18.62b	14.35±8.78c	70.80±10.34a	59.78±14.22ab	63.17±14.75a	-0.680**	-0.547**
	**Basidiomycota**	23.04±12.38b	75.88±14.89a	10.20±4.65c	19.29±12.32bc	8.77±3.27c	0.694**	0.612**
	Zygomycota	30.60±13.44a	9.34±10.59b	18.10±5.78ab	19.81±9.31ab	27.42±13.90a	-0.186	-0.218
	Chytridiomycota	0.56±0.30a	0.43±0.56a	0.89±0.56a	1.11±1.15a	0.63±0.47a	-0.104	-0.095

CK = fallow; X = vanilla monoculture; H = black pepper-vanilla rotation; B = pandan-vanilla rotation; C = sweet rice tea-vanilla rotation. Different letters denote a significant difference at the $P < 0.05$ level.

Table S7 Topological properties of co-occurrence networks within bacterial and fungal microbiomes

Network properties	CK	X	H	B	C
nodes_num	18	49	10	54	2
edges_num	9	45	5	75	1
positive.cor_num	5	32	3	72	1
negative.cor_num	4	13	2	3	0
average_degree	1.00	1.84	1.00	2.78	1.00
average_weight_degree	1.00	1.79	1.00	2.70	1.00
average_path_length	1.00	1.48	1.00	2.77	1.00
network_diameter	1.00	2.92	1.00	7.71	1.00
network_density	0.06	0.04	0.11	0.05	1.00
clustering_coefficient	NA	0.62	NA	0.55	NA
betweenness_centralization	0.00	0.00	0.00	0.06	NA
modularity	0.89	0.89	0.80	0.56	-0.50

CK = fallow; X = vanilla monoculture; H = black pepper-vanilla rotation; B = pandan-vanilla rotation;

C = sweet rice tea-vanilla rotation.
